# Estimating Asymptomatic and Symptomatic Transmission of the COVID‐19 First Few Cases in Selenge Province, Mongolia

**DOI:** 10.1111/irv.13277

**Published:** 2024-03-28

**Authors:** Davaalkham Dambadarjaa, Tsogt Mend, Andrew Shapiro, Mark S. Handcock, Undram Mandakh, Temuulen Enebish, Linh‐Vi Le, DJ Darwin R. Bandoy, Ambaselmaa Amarjargal, Bilegt Altangerel, Tuvshintur Chuluunbaatar, Uugantsetseg Guruuchin, Oyuntulkhuur Lkhagvajav, Oyunsuren Enebish

**Affiliations:** ^1^ School of Public Health Mongolian National University of Medical Sciences Ulaanbaatar Mongolia; ^2^ Department of Surveillance and Research National Center for Communicable Diseases Ulaanbaatar Mongolia; ^3^ Department of Statistics University of California Los Angeles California USA; ^4^ Department of Family Medicine, School of Medicine Mongolian National University of Medical Sciences Ulaanbaatar Mongolia; ^5^ COVID‐19 Incident Management Support Team World Health Organization Regional Office for the Western Pacific Manila Philippines; ^6^ College of Veterinary Medicine University of the Philippines Los Baños Laguna Philippines; ^7^ Ministry of Health Ulaanbaatar Mongolia

**Keywords:** Bayesian statistics, coronavirus infections/epidemiology*, COVID‐19*/epidemiology, family characteristics, field epidemiology, human*, influenza, pandemics*, SARS‐CoV‐2

## Abstract

**Background:**

Following the first locally transmitted case in Sukhbaatar soum, Selenge Province, we aimed to investigate the ultimate scale of the epidemic in the scenario of uninterrupted transmission.

**Methods:**

This was a prospective case study following the locally modified WHO FFX cases generic protocol. A rapid response team collected data from November 14 to 29, 2020. We created a stochastic process to draw many transmission chains from this greater distribution to better understand and make inferences regarding the outbreak under investigation.

**Results:**

The majority of the cases involved household transmissions (35, 52.2%), work transmissions (20, 29.9%), index (5, 7.5%), same apartment transmissions (2, 3.0%), school transmissions (2, 3.0%), and random contacts between individuals transmissions (1, 1.5%). The posterior means of the basic reproduction number of both the asymptomatic cases $R_{0}^{\mathrm{Asy}}$ and the presymptomatic cases $R_{0}^{\mathrm{Pre}}$ (1.35 [95% CrI 0.88–1.86] and 1.29 [95% CrI 0.67–2.10], respectively) were lower than that of the symptomatic cases (2.00 [95% Crl 1.38–2.76]).

**Conclusion:**

Our study highlights the heterogeneity of COVID‐19 transmission across different symptom statuses and underscores the importance of early identification and isolation of symptomatic cases in disease control. Our approach, which combines detailed contact tracing data with advanced statistical methods, can be applied to other infectious diseases, facilitating a more nuanced understanding of disease transmission dynamics.

## 1 Introduction

Although Mongolia is a neighboring country to China, it was able to delay domestic transmission of the COVID‐19 pandemic for an extended period. The country was one of the first to close its borders in response to the Wuhan outbreak in early 2020 and eventually closed schools and kindergartens, in addition to imposing travel restrictions and allowing only charter flights [1]. Initial measures, as directed by the State Emergency Committee, included increasing testing capacity, point of entry measures, quarantining incoming travelers in designated camps, canceling national holiday celebrations, banning mass gatherings, closing rail and land crossings, and restricting domestic travel. In response to vigorous public awareness campaigns and the promotion of protective measures, the public practiced social distancing and wearing masks extensively [2].

Having the foundation of the previously established ILI/SARI surveillance system throughout the country, the COVID‐19 surveillance system was implemented. The first national interim guideline for the surveillance and contact tracing of COVID‐19 was developed based on the WHO First Few Cases Investigation (FFX) Protocol in early 2020 [3, 4].

Despite aggressive preventive and risk‐reduction measures taken by the Government of Mongolia since the start of the pandemic, the first domestic transmission of COVID‐19 was confirmed by rt‐PCR in Ulaanbaatar city on November 10, 2020, followed by Sukhbaatar soum, Selenge Province on November 14, 2020, without any known link to the capital city [5].

Selenge Province is on the country's northern side, bordering Russia, with a total population of 108,370 and 29,359 households [6]. While strict border restrictions with China during 2020, the Altanbulag border checkpoint, only 24 km from Sukhbaatar soum, remained the only lifeline for returning land travelers and imported goods. During the first 2 months of the domestic transmission, the province was in complete lockdown, a total of 225 cases were confirmed out of 11,654 rt‐PCR tests, the test positivity rate was increased to 1.9, and the epidemic curve elevated to the peak at the second week and declined from fourth week in the province [7]. The country reported its first COVID‐19 death on December 26, 2020 [8].

In this study, we aimed to estimate the basic reproduction number based on the epidemiological and clinical characteristics of the first 67 confirmed cases of COVID‐19 in Sukhbaatar soum, Selenge Province, and to simulate the outbreak based on asymptomatic/symptomatic and presymptomatic profiles to determine the final size of the epidemic in the case of uninterrupted transmission.

## 2 Methods

### 2.1 Study Design

This was a prospective case study following the WHO FFX cases generic protocol with a few modifications based on the local context. The first 67 local COVID‐19 cases in Sukhbaatar soum, Selenge Province, Mongolia, were identified using a real‐time reverse transcription polymerase chain reaction (RT‐PCR) test. Once SARS‐CoV‐2 infection was confirmed, the local health department monitored the cases for up to 21 days until three consecutive negative RT‐PCR results were obtained. Their contacts were followed up for 14 days with three RT‐PCR tests. They were managed as confirmed cases according to local COVID‐19 regulations if the contact test results were positive. The rapid response teams used the national interim guidelines for COVID‐19 surveillance and the WHO FFX protocol questionnaire for data collection from confirmed cases and their close contacts. They followed the same procedure for confirmed cases if the RT‐PCR test results were positive for close contacts. The cycle threshold (Ct) value of the RT‐PCR tests was reportedly dependent on the period from infection and was useful for determining detectable RNA [9]. More specifically, as the Ct value reflects the viral load, the subjects with higher Ct‐values (Ct ≥ 30) are thought to be potentially less infectious. In particular, samples with Ct values ≥30 were no longer cultured and did not show detectable RNA [10]. Hence, during contact tracing, the infection potential lasted for approximately 2 weeks after contact, even in asymptomatic subjects.

### 2.2 Study Setting

**FIGURE 1:**
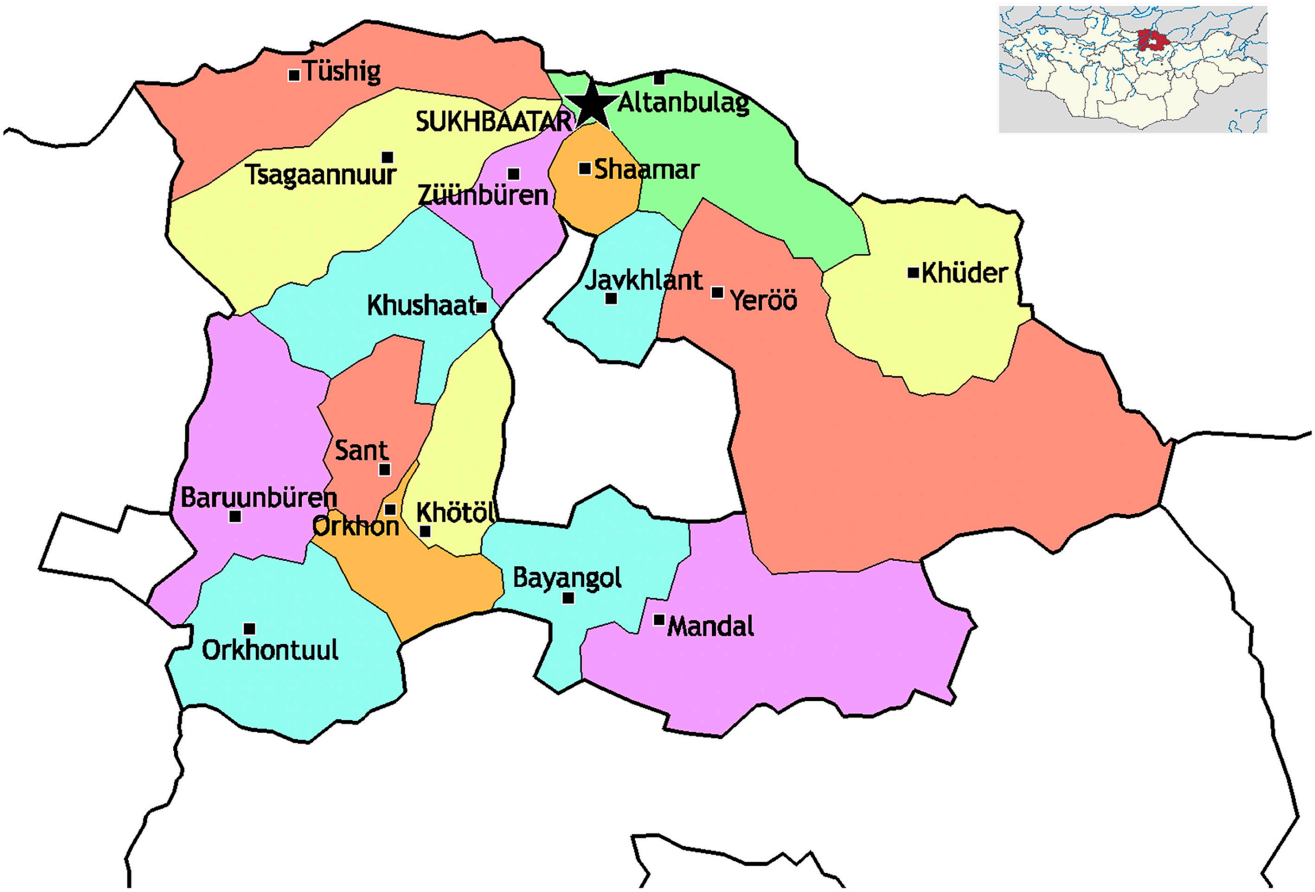
Sukhbaatar soum (denoted with a star) in the context of Selenge Province, Mongolia.

Selenge province has a railway station that connects to the Russian railway system with border crossing and checkpoint (Figure 1). Despite strict quarantine measures, a local case was reported without connection to the first locally transmitted case in Ulaanbaatar. Later, cases arose in the province, and local health administrative units and emergency agencies initiated rapid investigations and response teams to detect cases and their contacts. This action was followed by a strict lockdown, mandatory hospitalization of cases, and 21 days mandatory quarantine of close contacts. The emergency operation center deployed a rapid response team to alleviate in‐province health sector stress and workload. Until the domestic transmission, immediate testing capacity was not established at the provincial level. Specimens were sent to the closest province, Darkhan‐Uul and the virology laboratory of the National Center for Communicable Diseases (NCCD) for confirmation. Soon after the domestic transmission, the PCR laboratory capacity was built in the province.

### 2.3 Epidemiological and Laboratory Investigations

We collected data on the cases and their close contacts in Sukhbaatar soum, Selenge Province, from November 14 to 29, 2020. Field epidemiologists conducted either in‐person or remote interviews using mobile phones with cases and close contacts, collected demographic, travel history, epidemiological exposure, clinical symptoms, and potential risk factors for the infection transmission information based on local ethical considerations. Case definition was made according to the interim guideline for COVID‐19 surveillance and contact tracing. The hospital staff monitored the confirmed cases' and close contacts on a daily basis and contacts who developed symptoms were required to provide samples and be hospitalised if SARS‐CoV‐2 was detected by RT‐PCR.

### 2.4 Definition of Transmissions

Below, we provide definitions that guided the data‐cleaning procedures and were necessary for our modeling method. A confirmed case was a patient with a positive test result for SARS‐COV‐2 rt‐PCR. A case was considered active when transmissive, from inception until quarantine, recovery, non‐transmission, or death. A case is asymptomatic if the individual reported not developing symptoms from inception through recovery and was no longer transmissive. Asymptomatic transmission refers to the transmission of the virus from an active asymptomatic case to a secondary case. Presymptomatic transmission was the transmission of the virus from an active symptomatic patient to a secondary patient before the onset of symptoms. Symptomatic transmission refers to the transmission of the virus from an active symptomatic patient to a secondary patient after the onset of symptoms.

### 2.5 Data Cleaning

Each case in our dataset had a list of contact cases (all specified as not applicable [NA] if at the start of an outbreak), a date of symptom onset (NA if asymptomatic), a range of possible exposure dates, the end of their transmissible period (the earliest date between the date of quarantine, date of survey, or death), and a list of symptoms, if symptomatic. If a case had a date of onset but no listed symptoms (or minimal evidence of symptoms; this is at the discretion of the researcher), the patient was reclassified as asymptomatic, and the date of symptom onset was NA. If the date of symptom onset is incompatible with the range of exposure dates, occurring too soon or too late to be reasonable for the given pathogen, either the range of exposure dates was adjusted or the case was reclassified as asymptomatic, and the date of symptom onset was NA. If a secondary case's range of possible exposure dates was incompatible with the infecting case's range of possible exposure dates, the range of possible exposure dates for the two cases was adjusted, or the infecting case was removed from the secondary case's list of contact cases.

### 2.6 Stochastic Reconstruction of Transmission Chains

A transmission chain for the outbreak of a pandemic is a static network of confirmed cases, where network ties go from an infecting case to a secondary case, with each transmission occurring at a specific time and each case having a specific end in its transmissible period. In our dataset, some patients had multiple infectors and a wide range of possible exposure dates. This means that there was a greater distribution of all possible outbreaks that might have occurred, and a particular outbreak was one realization of that distribution. We created a stochastic process to draw many transmission chains from this greater distribution to better understand, and make inferences regarding, the outbreak under investigation. Specifics regarding this process and the assumptions underlying it are provided in Data S1.

### 2.7 Estimation of the Basic Reproductive Number ($R_{0}$)

For each realization of our stochastically reconstructed transmission chains, we estimated the posterior distributions of $R_{0}^{\mathrm{Asy}}$, $\;R_{0}^{\mathrm{Pre}}$, $R_{0}^{\mathrm{Sym}}$, and $R_{0}^{\mathrm{Tot}}$, the basic reproductive numbers of cases spread via asymptomatic, presymptomatic, symptomatic patients, and all transmissions. Each quantity was modelled using a Bayesian framework, a gamma prior [11], and COVID‐19 transmission was modelled with a Poisson process in time. Point estimates and the corresponding 95% credible intervals (CrI) were obtained from the posterior distributions using Monte Carlo integration. This method is an extension of that used by Cori et al. [12], leveraging our access to outbreak data as a transmission chain of cases instead of aggregate daily counts of cases to determine the reproductive number of subcategories of an outbreak in addition to the total reproductive number of an outbreak. All analyses were performed using R software (version 4.2.0) [13]. Additional details of this methodology are included in Data S1, including the software code used.

### 2.8 Predicting the Epidemic Outcomes Had Mitigation Not Been Applied

To better understand the potential impact of this outbreak in the absence of social distancing, quarantining, and other COVID‐19 transmission‐mitigating measures, we implemented a susceptible–infected–recovered (SIR) model that outputs estimates of the prevalence, total number of cases, incidence, number of new daily cases, and cumulative deaths. The SIR model was based on $R_{0}^{\mathrm{Tot}}$. In an SIR model, there is a fixed‐size population, and all members of the population are either susceptible (not infected but can be infected), infected (have the infection and can spread it to susceptible people), or recovered (had the infection but no longer have it and cannot spread it, nor can they get the infection again). We opted for an SIR model because the data collected only allowed us to understand the transmissions that occurred and not the network of contacts that did not result in new cases. As a result, we lacked information on exposure that could be credibly used in an SIR model for this outbreak. The details of this model are provided in Data S1.

## 3 Results

Between November 14 and 29, 2020, 67 patients were confirmed to have the SARS‐CoV‐2 infection. The index case in Selenge Province was detected on November 14, and 21 cases were identified through primary healthcare without any known connection to a confirmed or suspected case. The majority of the cases involved household transmissions (35, 52.2%), work transmissions (20, 29.9%), index (5, 7.5%), same apartment transmissions (2, 3.0%), school transmissions (2, 3.0%), and ad hoc meeting transmissions (1, 1.5%). This reveals that the home environment plays a critical role in the spread of SARS‐CoV‐2 infection, and understanding its dynamics is vital for implementing effective public health strategies.

Of the 67 patients, 38 (56.7%) were female, and the mean age was 36.1 (SD, 18.4). Regarding symptoms, of the 67 patients, 24 (35.8%) reported nasal congestion, 14 (23.3%) had a dry cough, 14 (22.6%) had a loss of smell and taste, and seven (11.5%) had a fever and other symptoms.

In terms of comorbidities, 10 (14.9%) patients were obese, 6 (9.0%) had renal disease, 4 (6.0%) had diabetes, 2 (3.0%) had cancer, and one case each (1.5%) of asthma and liver disease (Table 1).

**TABLE 1:** Characteristics of confirmed COVID‐19 cases in Sukhbaatar soum, Selenge Province, Mongolia, from November 14 to 29, 2020.

General characteristics of study participants	*n*	%
Sex		
Female	38	56.7%
Male	29	43.3%
Comorbidities		
Obesity	10	40.0%
Renal disease	6	24.0%
Liver disease	1	4.0%
Diabetes	4	16.0%
Cancer	2	8.0%
Heart disease	1	4.5%
Asthma	1	4.5%
Symptoms		
Nasal congestion	24	38.7%
Headache	15	23.8%
Dry cough	14	23.3%
Loss of smell and taste	14	22.6%
Fever	7	11.5%
Sore throat	7	11.3%
Runny nose	4	6.5%
Diarrhea	4	6.3%
Joint pain	4	6.3%
Nausea	4	6.3%
Shortness of breath	3	4.8%
Social status		
Employee	40	59.7%
Student	14	20.9%
Retired	10	14.9%
Unemployed	2	3.0%
Unknown	1	1.5%
Exposure		
Family	32	47.8%
Work	20	29.9%
Index	5	7.5%
Relative	3	4.5%
Lives in the same building	2	3.0%
School	2	3.0%
Unknown	2	3.0%
Ad hoc meeting	1	1.5%

Finally, we present in Table 2 the age distribution of cases compared to the age distribution of the population [6]. We can see the under representation of the youngest age group and the over representation of older age groups.

**TABLE 2:** Age distribution of the cases to that of the population of Selenge.

Age group	Confirmed cases	Proportion	Population	Proportion
0–9	4	6.00%	23,379	21.60%
10–19	10	14.90%	17,568	16.20%
20–29	13	19.40%	15,692	14.50%
30–39	11	16.40%	16,302	15.00%
40–49	12	17.90%	14,470	13.40%
50–59	10	14.90%	11,881	11.00%
≥60	7	10.40%	9078	8.40%
Total	67		108,370	

### 3.1 Transmission Chains

**FIGURE 2:**
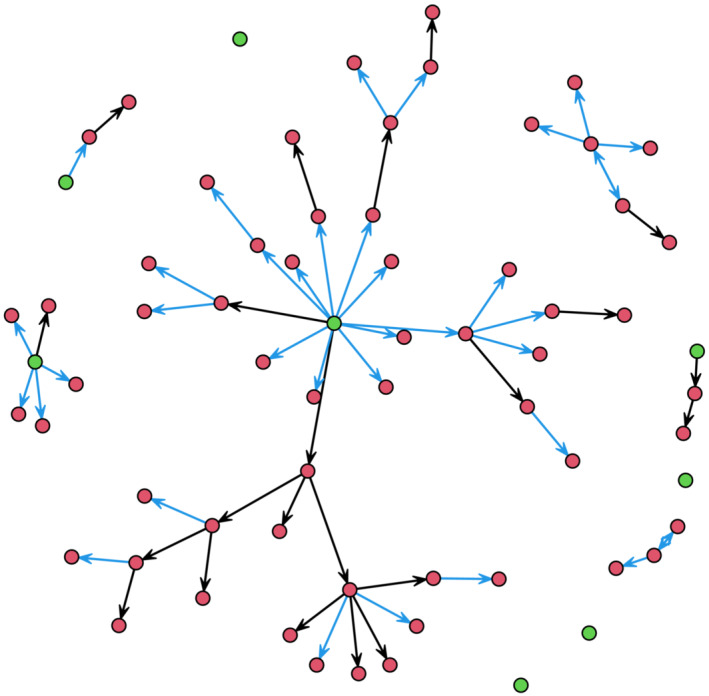
Transmission chains for the outbreak. The nodes are cases and the ties correspond to directed infection. If the direction of infection is ambiguous, ties are given in both directions. Green nodes are index cases. Blue edges correspond to infection from family members while black edges correspond to work or other non‐family infections.

Figure 2 graphically represents the transmission chains of the Sukhbaatar soum, Selenge Province outbreak.

Table 3 shows the distribution of the number of secondary infections per infection. Two‐thirds of the cases did not lead to additional infections. In addition, 13 secondary infections were associated with a single case. The mean number of secondary infections was 0.93 (SD 2.02).

**TABLE 3:** Distribution of the number of secondary infections per infection.

0	1	2	3	4	6	13
43	11	6	2	2	2	1

### 3.2 Estimation of $R_{0}$

**FIGURE 3:**
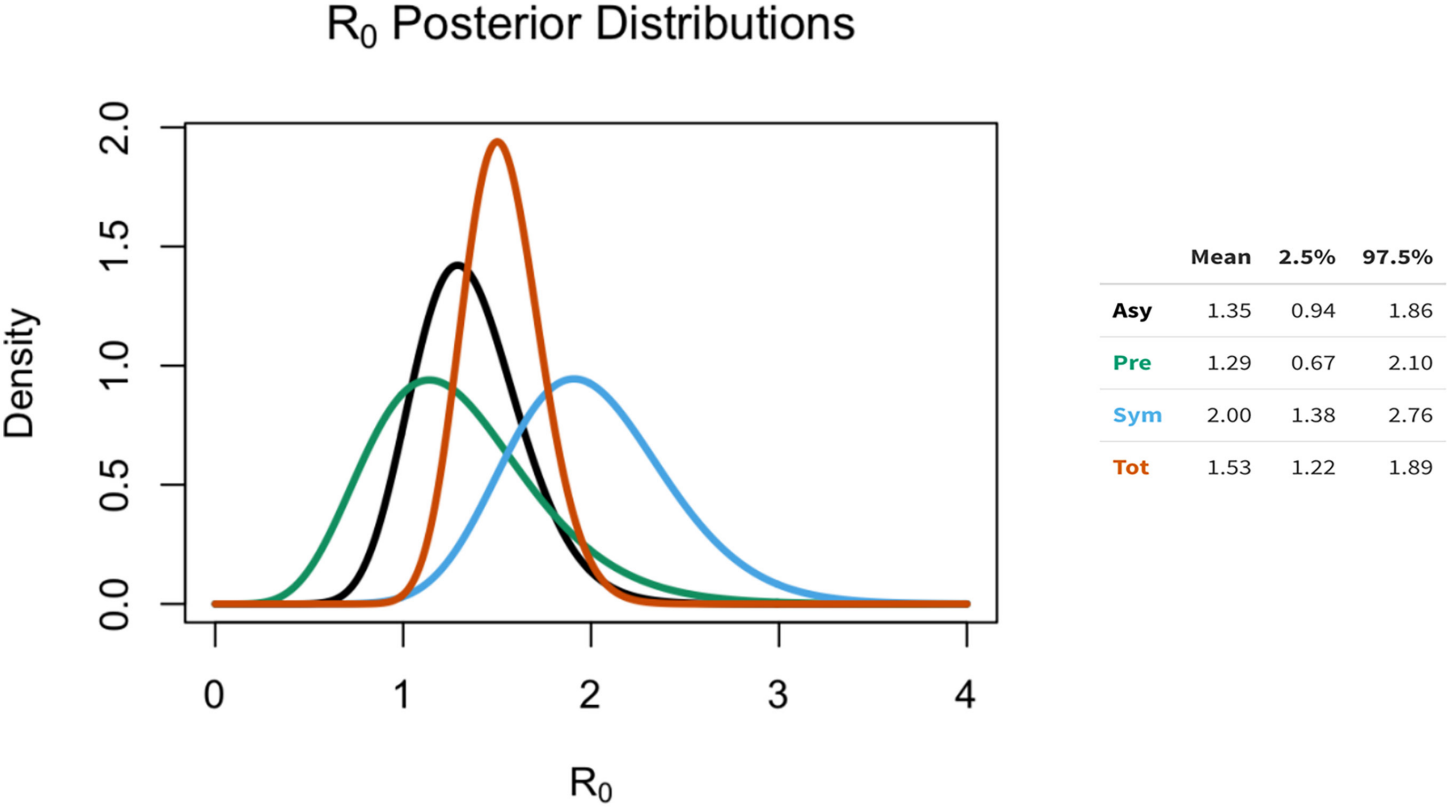
Estimates of the reproductive numbers for three types of transmission (**asy**mptomatic, presymptomatic, and symptomatic). Each distribution summarizes the knowledge we have about the corresponding reproductive number and the relative probability of that value. More concentrated distributions represent more precise knowledge, based on the study.

Figure 3 shows the estimates of the symptomatic, asymptomatic, presymptomatic, and total reproductive numbers of the Selenge outbreak.

The posterior means of the basic reproductive number for symptomatic cases, $R_{0}^{\mathrm{Sym}},$ was 2.00 (95% CrI 1.38–2.76), indicating that individuals displaying symptoms of the disease have a higher transmission potential compared to asymptomatic and presymptomatic cases. Notably, the posterior means of the basic reproduction number of both the asymptomatic cases, $R_{0}^{\mathrm{Asy}}$ and presymptomatic cases $R_{0}^{\mathrm{Pre}}$(1.35 [95% CrI 0.88–1.86] and 1.29 [95% CrI 0.67–2.10], respectively), were lower than that of the symptomatic cases (2.00 [95% Crl 1.38–2.76]).

This suggests that individuals who have not yet developed symptoms but are infected can also transmit the disease to others. Therefore, this resulted in an aggregated $R_{0}^{\mathrm{Tot}}$ value of 1.53 (95% CrI 1.22–1.89) (Figure 3).

### 3.3 Estimation of Potential Epidemic Size and Impact

**FIGURE 4:**
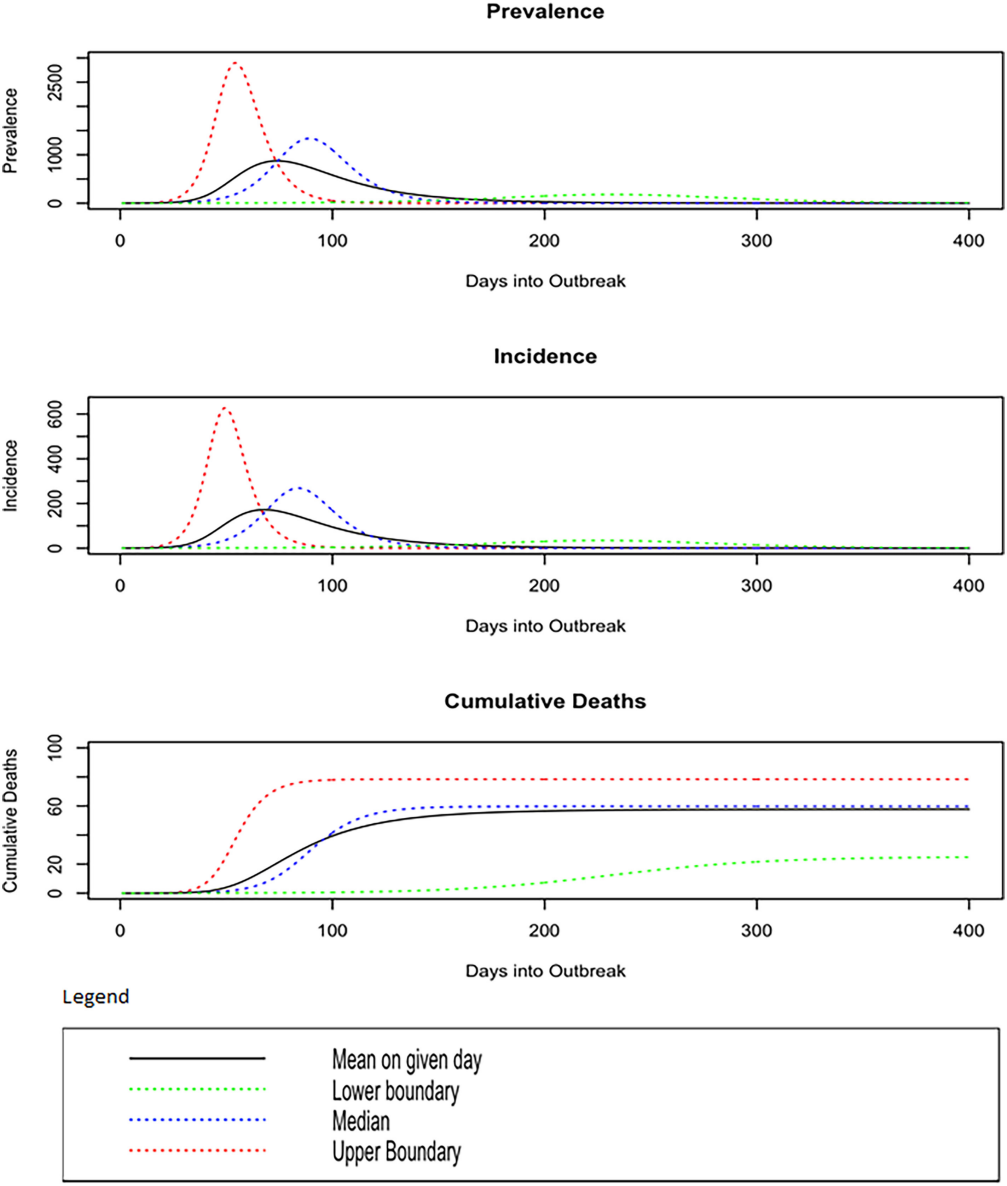
Estimates of the prevalence, incidence, and cumulative deaths over time had mitigation not occurred. The lower/upper boundaries are the 2.5%/97.5% probability events. The difference between the mean and median outcomes reflects the skewness of the distributions.

The SIR model was run with the starting conditions of *S*(0) = 20,000, *I*(0) = 1, and *R*(0) = 0 to mimic the conditions of the outbreak in Sukhbaatar soum, where a single infection that started the outbreak in a soum with a population of approximately 20,000. COVID‐19 was modeled with a mean infectious time of 1/𝛾 = 5.22, and a case fatality rate of 0.05 [14, 15]. Figure 4 includes estimates of the mean Prevalence, Incidence, and Cumulative Deaths according to the number of days since the outbreak (the solid black line). We also included estimates of Prevalence, Incidence, and Cumulative Deaths for a lower boundary value (the 2.5% percentile of our sample of $R_{0}^{\mathrm{Tot}}$), an upper boundary value (the 97.5 quantile of our sample of $R_{0}^{\mathrm{Tot}}$), and a highly probable value (the median of our sample) of $R_{0}^{\mathrm{Tot}}$ to give reasonable expectations of what an outbreak might have looked like in best case, worst case, and realistic case scenarios. An important caveat is that the SIR model is run with a fixed case fatality rate. The health care facilities of a small soum of 20,000 would likely be overwhelmed by a prevalence of 3000 cases, resulting in infected individuals (1) not receiving adequate care and (2) a higher case fatality rate. Therefore, for the worst‐case scenarios, it is reasonable to assume that the Cumulative Deaths are underestimated, and the effect of the outbreak in a worst‐case scenario could be far more severe. It is worth comparing the observed numbers of cases and deaths to these scenarios. All 67 cases occurred within the first 25 days of the outbreak and there were no deaths. These are not grossly different from the scenarios. However, under the scenarios the epidemic size and impact after the first 25 days are much larger than that observed, indicating the strong effect of the public health intervention.

## 4 Discussion

Our study found that symptomatic individuals had a higher basic reproduction number compared to asymptomatic and presymptomatic individuals. This discovery underscores the potential for symptomatic individuals to be significant drivers of COVID‐19 transmission. However, the reproductive number for presymptomatic individuals was lower than that found in a similar study conducted by Seyed et al. [16]. The lower reproduction number in our study could be attributed to the stringent public health and social measures implemented in Sukhbaatar soum, Selenge province during our study period. We posit that the early case finding and contact tracing initiatives carried out in our study significantly helped minimize possible larger transmissions in this rural setting. Moreover, multi‐sectoral collaboration coordinated according to the incident management system and within this commitment, rapid response teams might have had a significant role in this outbreak.

At the beginning of the pandemic and before the availability of vaccines, most countries with emerging cases limited the spread of COVID‐19 through the strict closure of schools, public services, and organizations [17, 18]. The efforts to contain the disease and maintain the pathogen reproductive number R_t_ at <1 placed a major strain on economies and societies [19, 20]. Nevertheless, some countries managed to successfully control transmission without imposing a mandatory lockdown. A notable example is Taiwan, which, despite its close proximity to China, had among the lowest COVID‐19 incidence and mortality rates globally [21]. Population‐based measures such as face mask use, social distancing, maintaining hygiene, and case‐based strategies, including case detection, contact tracing, quarantine, and surveillance, were used to adequately decrease COVID‐19 transmission [22]. Contact tracing aims to detect possible cases that were in contact with a newly identified COVID‐19 patient. However, unless contact tracing capabilities are sufficient, the efficacy is reduced, mostly because of presymptomatic cases or delays between symptom onset and detection [23]. In another example, Rwanda was one of the first countries in Africa to take action against COVID‐19 transmission by screening all passengers from countries with confirmed cases and implementing a countrywide lockdown. The country rigorously utilizes contact tracing for early detection, and uses data to estimate secondary attack rates and spatial analysis to determine high‐risk areas [24].

The United Kingdom was one of the first European countries to have new emerging cases. While most cases were imported, most secondary cases were close contacts [25]. In a single‐center retrospective analysis of the first 500 confirmed cases in Manila, the Philippines, 133 (26.6%) were healthcare workers (HCW), and 367 (73.4%) were non‐HCW [26]. Similarly, in Vietnam, among the first few hundred confirmed cases, 60% were imported and 43% of cases remained asymptomatic for the duration of infection [27]. Results of previously published work on nationwide sero‐prevalence of SARS‐COV‐2 in Mongolia showed that sero‐positivity was associated with symptomatic cases and higher hospitalization rates [28, 29].

For the current study, we used the locally modified version of the first few cases and contacts transmission investigation protocol, one of the UNITY initiatives [4, 30]. Despite facing technical challenges like study quality and communication issues, investigators from low‐ and middle‐income countries widely acknowledged the initiative's pivotal role in establishing equal research opportunities and promoting collaboration, with substantial support from the World Health Organization in terms of technical assistance, serological assays, and funding for study implementation [31]. In the Mongolian context, following the International Health Regulation (IHR) and Asia Pacific Strategy for Emerging Diseases and Public Health Emergency (APSED) [32], Mongolia had previously implemented an incident management system (IMS) with comprehensive documentation for disaster and public health emergency management, overseen by the Deputy Prime Minister. The advent of the COVID‐19 pandemic brought both advantages and disadvantages to light, prompting the development of an enhanced version in 2023 [33]. Regular multi‐sectoral simulation exercises are stated as one of the components.

Despite robust individual case interviews and contact‐tracing records, missing data was the main challenge in this study. In the future, developing more country‐specific preplanned FFX to improve pandemic preparedness tailors public health and social measures around the evidence generated [34]. The local data collection capacity should also be modernized to assure data quality. Including a training module in the field epidemiologists training program (FETP) regarding the locally adapted FFX approach for data collection and contact tracing is strongly suggested.

Moreover, to avoid challenges and barriers encountered in our study, countries should integrate and digitalize health information systems (HIS) to ensure long‐term possible pandemic preparedness and readiness. Resiliency should be carefully adapted to the local health systems to avoid socio‐economic impacts caused by public health and social measures. These can be avoided by a rapid response mechanism with quality‐ensured epidemiological and other relevant sector data. Despite reasonable multi‐sectoral collaborative efforts, the lack of digitalization and modernization of data collection was the root of the problem for further investigation and analysis.

Our model used a common relative infectivity profile for all individuals. The relative infectivity profile may vary according to an individual's age and other characteristics. However, there is insufficient knowledge of the infectivity profiles to allow for this level of detail.

An important contribution of this work is the creation of open‐source software that implements novel statistical methods. This software was written in the open‐source R^13^ statistical language and is available in Supporting Information.

Our approach can be applied not only to other variants of SARS‐CoV‐2 but also to other infectious diseases. Understanding the varying reproductive numbers among different groups can enhance contact tracing strategies and public health messaging for emerging infectious diseases. Future research should investigate how these findings can be applied across different pathogens and epidemiological contexts.

## 5 Conclusion

Our findings provide new insights into the reproductive numbers among symptomatic, asymptomatic, and presymptomatic individuals, and underscore the importance of robust public health measures and advanced data management systems in controlling infectious disease transmission.

## Author Contributions

**Davaalkham Dambadarjaa:** Conceptualization (supporting); Validation (equal); Writing – original draft Preparation (equal). **Tsogt Mend:** Conceptualization (supporting); Data curation (equal); Formal analysis (equal); Validation (equal); Visualization (equal); Writing – original draft (equal); Writing – review and editing (equal). **Andrew Shapiro:** Conceptualization (supporting); Formal analysis (equal); Methodology (equal); Visualization (equal); Writing – original draft (equal). **Mark S. Handcock:** Conceptualization (lead); Supervision (equal); Methodology (equal); Resources (equal); Validation (equal); Writing – review and editing (equal). **Undram Mandakh:** Conceptualization (supporting); Supervision (equal); Funding acquisition (lead); Project Administration (equal); Writing – original draft (equal); Writing – review and editing (equal). **Temuulen Enebish:** Validation (supporting); Project administration (supporting); Writing – review and editing (equal). **Linh‐Vi Le:** Conceptualization (supporting); Methodology (equal); Project administration (equal); Writing – review and editing (equal). **DJ Darwin R. Bandoy:** Validation (equal); Writing – review and editing (equal). **Ambaselmaa Amarjargal:** Project administration (equal); Resources (equal). **Bilegt Altangerel:** Investigation (equal). **Tuvshintur Chuluunbaatar:** Investigation (equal). **Uugantsetseg Guruuchin:** Investigation (equal). **Oyuntulkhuur Lkhagvajav:** Investigation (equal). **Oyunsuren Enebish:** Funding acquisition (equal); Project administration (supporting); Resources (equal).

## Ethics Statement

This study was undertaken as part of the national pandemic response and received MoH Ethical clearance on February 2, 2021, with Institutional Review Board Approval No. 202.

## Conflicts of Interest

The authors declare no conflicts of interest.

### Peer Review

The peer review history for this article is available at https://www.webofscience.com/api/gateway/wos/peer‐review/10.1111/irv.13277.

## Supporting information


**Figure S1.** Gamma distribution used for the estimation.
**Figure S2.** Gamma distribution for symptomatic cases.

## Data Availability

The data that support the findings of this study are available on request from the corresponding author. The data are not publicly available due to privacy or ethical restrictions.

## References

[1] R. Erkhembayar , E. Dickinson , D. Badarch , et al., “Early Policy Actions and Emergency Response to the COVID‐19 Pandemic in Mongolia: Experiences and Challenges,” The Lancet Global Health 8, no. 9 (2020): e1234–e1241, 10.1016/S2214-109X(20)30295-3. 32711684 PMC7377809

[2] A. Dagvadorj , B. Jantsansengee , O. O. Balogun , T. Baasankhuu , and B. Lkhagvaa , “Health Emergency Preparedness and Response to the COVID‐19 Pandemic: Lessons Learnt From Mongolia,” The Lancet Regional Health–Western Pacific 21 (2022): 100436, 10.1016/j.lanwpc.2022.100436. 35350463 PMC8948500

[3] Ministry of Health, Mongolia , “Health Ministerial Order no.269: Interim Guideline for COVID‐19 Surveillance, Prevention and Rapid Response,” published online April 29, 2020, https://moh.gov.mn/uploads/files/c5ece091d53498a44209f967428c4acb0eaecd2d.pdf.

[4] World Health Organisation , “The UNITY Studies: WHO Sero‐Epidemiological Investigations Protocols,” accessed June 24, 2022, https://www.who.int/emergencies/diseases/novel‐coronavirus‐2019/technical‐guidance/early‐investigations.

[5] WHO Mongolia Country Office , “Coronavirus Disease 2019 (COVID‐19) Situation Report #29,” accessed June 20, 2022, https://www.who.int/docs/default‐source/wpro‐‐‐documents/countries/mongolia/covid‐19/sitrep‐29‐2020‐11‐22.pdf?sfvrsn=eb6747cd_2.

[6] National Statistics Office , “Population of Mongolia,” published online 2022, accessed November 25, 2023, https://www.1212.mn/en/statistic/statcate/573051/table‐view/DT_NSO_0300_006V1.

[7] WHO Mongolia Country Office , “Annual Report 2020 for Multi Partner COVID‐19 Response and Recovery Fund”.

[8] Multi sectoral Emergency Operation Center , “COVID‐19 Daily Situation Report December 26, 2020”.

[9] J. Bullard , K. Dust , D. Funk , et al., “Predicting Infectious Severe Acute Respiratory Syndrome Coronavirus 2 From Diagnostic Samples,” Clinical Infectious Diseases 71, no. 10 (2020): 2663–2666, 10.1093/cid/ciaa638. 32442256 PMC7314198

[10] A. Singanayagam , M. Patel , A. Charlett , J. Lopez Bernal , V. Saliba , J. Ellis , S. Ladhani , M. Zambon , and R. Gopal . “Duration of Infectiousness and Correlation With RT‐PCR Cycle Threshold Values in Cases of COVID‐19, England, January to May 2020,” Eurosurveillance 25, no. 32 (2020): 2001483, 10.2807/1560-7917.ES.2020.25.32.2001483. 32794447 PMC7427302

[11] Z. Zhuang , S. Zhao , Q. Lin , et al., “Preliminary Estimation of the Novel Coronavirus Disease (COVID‐19) Cases in Iran: A Modelling Analysis Based on Overseas Cases and Air Travel Data,” International Journal of Infectious Diseases 94 (2020): 29–31, 10.1016/j.ijid.2020.03.019. 32171951 PMC7194910

[12] A. Cori , N. M. Ferguson , C. Fraser , and S. Cauchemez , “A New Framework and Software to Estimate Time‐Varying Reproduction Numbers During Epidemics,” American Journal of Epidemiology 178, no. 9 (2013): 1505–1512, 10.1093/aje/kwt133. 24043437 PMC3816335

[13] R Core Team , “R: A Language and Environment for Statistical Computing,” published online 2018, https://www.R‐project.org/.

[14] X. He , E. H. Y. Lau , P. Wu , et al., “Temporal Dynamics in Viral Shedding and Transmissibility of COVID‐19,” Nature Medicine 26, no. 5 (2020): 672–675, 10.1038/s41591-020-0869-5. 32296168

[15] The World Health Organization , “Estimating Mortality From COVID‐19,” https://www.who.int/publications‐detail‐redirect/WHO‐2019‐nCoV‐Sci‐Brief‐Mortality‐2020.1.

[16] S. M. Moghadas , M. C. Fitzpatrick , P. Sah , et al., “The Implications of Silent Transmission for the Control of COVID‐19 Outbreaks,” Proceedings of the National Academy of Sciences 117, no. 30 (2020): 17513–17515, 10.1073/pnas.2008373117. PMC7395516 32632012

[17] J. M. Brauner , S. Mindermann , M. Sharma , et al., “Inferring the Effectiveness of Government Interventions Against COVID‐19,” Science 371, no. 6531 (2021): eabd9338, 10.1126/science.abd9338. 33323424 PMC7877495

[18] J. Dehning , J. Zierenberg , F. P. Spitzner , et al., “Inferring Change Points in the Spread of COVID‐19 Reveals the Effectiveness of Interventions,” Science 369, no. 6500 (2020): eabb9789, 10.1126/science.abb9789. 32414780 PMC7239331

[19] A. Byambadorj , S. Amarsanaa , O. Enebish , et al., “Spending Assessment and Economic Burden of COVID‐19 in Mongolia, January‐ September, 2020,” Asia‐Pacific Journal of Public Health 34, no. 4 (2022): 456–458, 10.1177/10105395221074551. 35086346

[20] B. Weder di Mauro and R. Baldwin , Economics in the Time of COVID‐19 (Paris & London: CEPR Press, 2020), https://cepr.org/publications/books‐and‐reports/economics‐time‐covid‐19.

[21] T. C. Ng , H. Y. Cheng , H. H. Chang , et al., “Comparison of Estimated Effectiveness of Case‐Based and Population‐Based Interventions on COVID‐19 Containment in Taiwan,” JAMA Internal Medicine 181, no. 7 (2021): 913–921, 10.1001/jamainternmed.2021.1644. 33821922 PMC8025126

[22] H. Y. Cheng and A. S. E. Huang , “Proactive and Blended Approach for COVID‐19 Control in Taiwan,” Biochemical and Biophysical Research Communications 538 (2021): 238–243, 10.1016/j.bbrc.2020.10.100. 33220926 PMC7831726

[23] B. J. Gardner and A. M. Kilpatrick , “Contact Tracing Efficiency, Transmission Heterogeneity, and Accelerating COVID‐19 Epidemics,” PLoS Computational Biology 17, no. 6 (2021): e1009122, 10.1371/journal.pcbi.1009122. 34138866 PMC8241027

[24] M. Semakula , F. Niragire , A. Umutoni , et al., “The Secondary Transmission Pattern of COVID‐19 Based on Contact Tracing in Rwanda,” BMJ Global Health 6, no. 6 (2021): e004885, 10.1136/bmjgh-2020-004885. PMC8189754 34103325

[25] N. L. Boddington , A. Charlett , S. Elgohari , et al., “Epidemiological and Clinical Characteristics of Early COVID‐19 Cases, United Kingdom of Great Britain and Northern Ireland,” Bulletin of the World Health Organization 99, no. 3 (2021): 178–189, 10.2471/BLT.20.265603. 33716340 PMC7941108

[26] K. A. Agrupis , C. Smith , S. Suzuki , et al., “Epidemiological and Clinical Characteristics of the First 500 Confirmed COVID‐19 Inpatients in a Tertiary Infectious Disease Referral Hospital in Manila, Philippines,” Tropical Medicine and Health 49, no. 1 (2021): 48, 10.1186/s41182-021-00340-0. 34118992 PMC8196293

[27] P. Q. Thai , M. A. Rabaa , D. H. Luong , et al., “The First 100 Days of Severe Acute Respiratory Syndrome Coronavirus 2 (SARS‐CoV‐2) Control in Vietnam,” Clinical Infectious Diseases 72, no. 9 (2021): e334–e342, 10.1093/cid/ciaa1130. 32738143 PMC7454342

[28] B. Chimeddorj , U. Mandakh , L. V. le , et al., “SARS‐CoV‐2 Seroprevalence in Mongolia: Results From a National Population Survey,” The Lancet Regional Health–Western Pacific 17 (2021): 100317, 10.1016/j.lanwpc.2021.100317. 34841381 PMC8609908

[29] B. Chimeddorj , C. R. Bailie , U. Mandakh , et al., “SARS‐CoV‐2 Seroepidemiology in Mongolia, 2020–2021: A Longitudinal National Study,” The Lancet Regional Health–Western Pacific 36 (2023): 100760, 10.1016/j.lanwpc.2023.100760. 37360871 PMC10084888

[30] I. Bergeri , H. C. Lewis , L. Subissi , et al., “Early Epidemiological Investigations: World Health Organization UNITY Protocols Provide a Standardized and Timely International Investigation Framework During the COVID‐19 Pandemic,” Influenza and Other Respiratory Viruses 16, no. 1 (2022): 7–13, 10.1111/irv.12915. 34611986 PMC8652791

[31] K. Hennessey , L. Pezzoli , and C. Mantel , “A Framework for Seroepidemiologic Investigations in Future Pandemics: Insights From an Evaluation of WHO's UNITY Studies Initiative,” Health Research Policy and Systems 21, no. 1 (2023): 34, 10.1186/s12961-023-00973-z. 37194007 PMC10187500

[32] “Asia Pacific Strategy for Emerging Diseases and Public Health Emergencies (APSED III): Advancing Implementation of the International Health Regulations (2005): Working Together Towards Health Security,” published online September 18, 2017, accessed November 30, 2023, https://iris.who.int/bitstream/handle/10665/259094/9789290618171‐eng.pdf?sequence=1.

[33] “Regulation on Communicating All‐Hazard Multi‐Source Surveillance Information for Early Warning of Potential Disasters and Public Health Emergencies Across Different Sectors,” Deputy Prime Minister's order No.28, 2023.

[34] H. C. Lewis , A. J. Marcato , N. Meagher , et al., “Transmission of SARS‐CoV‐2 in Standardised First Few X Cases and Household Transmission Investigations: A Systematic Review and Meta‐Analysis,” Influenza and Other Respiratory Viruses 16, no. 5 (2022): 803–819, 10.1111/irv.13002. 36825117 PMC9343340

