# Supplementary material for: Estimating Asymptomatic and Symptomatic Transmission of the COVID‐19 First Few Cases in Selenge Province, Mongolia
Source: Influenza Other Respir Viruses. 2024 Mar 28;18(4):e13277. doi: 10.1111/irv.13277 (PMC10973774; doi:10.1111/irv.13277)
Supplement: Supplementary file 1 — Figure S1. Gamma distribution used for the estimation. Figure S2. Gamma distribution for symptomatic cases. [file IRV-18-e13277-s001.pdf]

Supporting Information to

"Estimating asymptomatic and symptomatic transmission of the COVID-19 first few cases in Selenge province, Mongolia"

by

Andrew Shapiro, Mark S. Handcock
Department of Statistics, University of California, Los Angeles, USA

Stochastic Reconstruction of Transmission Chains

For each case, we knew the date of symptom onset (for symptomatic cases), the date of quarantining (the date when a confirmed positive case enters quarantining and can no longer spread COVID-19 to others), each case's infector or possible infectors (in our data, some cases had as many as three contacts with confirmed positive cases that could have been the infector for the given cases; for cases with a single contact, that contact is assumed to be the infector), and the range of dates of possible incidence of each case. Additionally, we have infectivity profiles $P_{Inf}^{Asy}(s)$, $P_{Inf}^{Pre}(s)$, and $P_{Inf}^{Sym}(s)$, which are the probability distributions dependent on time, s, and independent of calendar time, for asymptomatic, presymptomatic, and symptomatic cases, respectively. The infectivity profiles for asymptomatic, presymptomatic, and symptomatic cases were those modeled from 77 transmission pairs obtained from publicly available sources within and outside mainland China (He et.al. 2020) and are detailed in the end section below.

Other methods of transmissivity modeling (Cori et. al. 2013) use the serial distribution, or the number of days between days of symptom onset between an infector and infectee case. This method works well for pathogens that are largely symptomatic. The large number of asymptomatic cases for COVID-19 makes it unreasonable to use the serial intervals, and instead we must use the generation interval, or the number of days between the incidence of a case in the infector and the incidence of a case in the infectee. This poses an additional problem since the time of incidence is virtually impossible to witness. To overcome this, we can use the data we have to stochastically reconstruct the transmission chains of the COVID-19 breakout and effectively Monte Carlo integrate out the times of incidence to model infectivity. This also provides the additional benefit of being able to model transmissivity of different types of transmission, more specifically, but not limited to, asymptomatic and symptomatic transmission.

To create these stochastically reconstructed transmission chains, we assume the infections are independent conditional on the set of infectors. We also assume that each potential infector within each class has the same infectivity profile. For each case i and each possible infector j, we determine the range of viable days j could have infected i, t_j. These days need not be contiguous. Then, according to the infectivity profile, the probability that j infected i on the kth day of t_j is $P_{Inf}^{A_{j,k}}\left(t_{jk}\right)$, where $A\in\{Asy, Pre,\ Sym\}$ is the symptomatic class of j on day k. Then the probability that j infected i during t_j is

$$P_j = \sum_{k=1}^{\left|t_j\right|} P_{Inf}^{A_{j,k}}\left(t_{jk}\right)$$

for each $j\in J$, the set of all possible infectors of i. We can then reweight $\hat{P}_j = P_j / \sum_{j\in J} P_j$, then make a simple random draw from $\hat{P}$ to determine which j infected i in our reconstructed chain and make a simple random draw from $P_{Inf}^{A_{j,k}}\left(t_j\right)$ to determine which day j infected i. Note, the date of incident of the infector case will affect the viable range of dates the infectee case could have been infected on, so all possible infector cases must have their incident date drawn before drawing an infectee's incident date. We then continue to any further links in the chains conditioning on prior events. This process produces a single reconstructed transmission chain. We then repeat the complete process to sample additional chains.

Estimation of the basic reproductive number (R_0)

For each realization of our stochastically reconstructed transmission chains, we estimate posterior distributions of R_0^{Asy},R_0^{Sym},R_0^{Tot}, the basic reproductive numbers of asymptomatic cases, symptomatic cases, and of all cases. We modeled transmission between individuals with a Poisson process in time, so that the instantaneous rate at which a case became infected or had symptom onset at time $t - s$ is $R_0^A P_{Inf}^A(t - s)$, $A\in\{Asy, Pre, Sym, Tot\}$. We assume these infection times are independent. Hence, Y_t^A, the number of people infected at time t from cases of symptomatic class A, is Poisson with mean $R_0^A \sum_{s=0}^{t-1} Y_s^A P_{Inf}^A(t - s)$. The instantaneous reproduction number for each symptomatic class A is assumed to be constant at R_0^A throughout the study. The likelihood of Y_t^A given R_0^A and $Y_0^A, \ldots, Y_{t-1}^A$ is then

$$P\left(Y_t^A | R_0^A, Y_0^A, ..., Y_{t-1}^A, P_{Inf}^A\right) = \frac{\left(R_0^A \sum_{s=0}^{t-1} Y_s^A P_{Inf}^A(t-s)\right)^{Y_t^A} exp\left(-R_0^A \sum_{s=0}^{t-1} Y_s^A P_{Inf}^A(t-s)\right)}{Y_t^A!}$$

For a given outbreak, each case i has a start time s_i^A, and end time e_i^A during which it was of symptomatic class A. Note, if a case was never of a particular class, $s_i^A = e_i^A = 0$, but for cases that transition between classes, in this case from presymptomatic to symptomatic, then $s_i^{Pre} = 0$, $e_i^{Pre} = t_i^{onset} - 1$, and $s_i^{Sym} = t_i^{onset}$, $e_i^{Sym} = t_i^{quarantine}$. We define the infectivity mass at time t of symptomatic class A as:

$$M_t^A = \sum_{s=0}^{t} Y_s^A P_{Inf}^A(t - s).$$

Similarly, we define the infectivity mass of case i for symptomatic class A as:

$$M_i^A = \sum_{j=s_i^A}^{e_i^A} P_{Inf}^A(j).$$

For an outbreak that occurs during $\left[t_{start}, t_{end}\right]$ and has cases $i = 1, ..., n$ for class A,

$$\sum_{t=t_{start}}^{t_{end}} M_t^A = \sum_{i=1}^{n} M_i^A.$$

We can simplify the likelihood of Y_t^A given R_0^A and $Y_0^A, ..., Y_{t-1}^A$ as

$$P\left(Y_t^A | R_0^A, Y_0^A, ..., Y_{t-1}^A, P_{Inf}^A\right) = \frac{\left(R_0^A M_t^A\right)^{Y_t^A} exp\left(-R_0^A M_t^A\right)}{Y_t^A!}.$$

Since R_0^A is considered to be constant over the course of the outbreak, the likelihood of transmission over $[t - \tau + 1, t]$ by cases of symptomatic class A, $Y_{t-\tau+1}^A, ..., Y_t^A$, given R_0^A and $Y_0^A, ..., Y_{t-\tau}^A$ is

$$P(Y_{t-\tau+1}^A, ..., Y_t^A \mid Y_0^A, ..., Y_{t-\tau}^A, R_0^A, P_{Inf}^A) = \prod_{s=t-\tau+1}^{t} \frac{\left(R_0^A M_t^A\right)^{Y_s^A} exp\left(-R_0^A M_t^A\right)}{Y_s^A!}$$

If we give R_0^A a prior distribution of $\Gamma(shape = a, rate = b)$, then under a Bayesian framework, the joint posterior distribution of R_0^A is

$$P\left(Y^A_{t-\tau+1},\dots, Y^A_t, R^A_0 | Y^A_0,\dots, Y^A_{t-\tau}, P^A_{Inf}\right) = P\left(Y^A_{t-\tau+1},\dots, Y^A_t | Y^A_0,\dots, Y^A_{t-\tau}, R^A_0, P^A_{Inf}\right) P\left(R^A_0\right) =$$

$$\prod_{s=t-\tau+1}^{t} \frac{\left(R^A_0 M^A_s\right)^{Y^A_s} e^{-R^A_0 M^A_s}}{Y^A_s!} \frac{\left(R^A_0\right)^{a-1} e^{-R^A_0 b} b^a}{\Gamma(a)}$$

$$= \left(R^A_0\right)^{a-1+\sum_{s=t-\tau+1}^{t} Y_t} e^{-R^A_0\left(b+\sum_{s=t-\tau+1}^{t} M^A_t\right)} \prod_{s=t-\tau+1}^{t} \frac{b^a \left(M^A_t\right)^{Y_t}}{\Gamma(a) Y_t!}.$$

Hence, the posterior distribution of R_0^A is $\Gamma\left(a + \sum_{s=t-\tau+1}^{t} Y^A_t, b + \sum_{s=t-\tau+1}^{t} M^A_t\right)$. Thus, over the entirety of the outbreak, with cases $1,\dots, n$, and Y^a_i being the number of cases infected by i while of symptomatic class A,

$$R^A_0 \sim \Gamma\left(shape = a + \sum_{i=1}^{n} Y^A_i, rate = b + \sum_{i=1}^{n} M^A_i\right).$$

We estimated R_0^{Asy}, R_0^{Pre}, R_0^{Sym}, and R_0^{Tot} separately. The prior distribution for each R_0^A is Gamma with mean 2.5 and standard deviation 2 (corresponds with $shape = 1.25$ and $rate = 0.625$), expressing large uncertainty about the basic reproductive number in this context (Zhang et.al. 2020). For each chain, we calculate the realized total number of people infected from case i while of symptomatic class A, y^A_i, and the infectivity mass of each case for each symptomatic class A, m^A_i. The posterior distribution results in

$$R^A_0 \sim \Gamma\left(shape = 1.25 + \sum_{i=1}^{n} y^A_i, rate = 0.625 + \sum_{i=1}^{n} m^A_i\right),$$

for n total cases in the outbreak. The posterior distribution can be computed directly for asymptomatic, presymptomatic, symptomatic, and all cases separately.

All analyses were done using R software (version 4.2.0). All quantities were estimated in a Bayesian framework. Point estimates and the corresponding 95% credible intervals (CrI) were obtained from the posterior distributions.

Assumed infectiousness profiles of COVID-19 used to estimate the reproductive number

The infectivity profiles $P_{Inf}^{Asy}(s)$, $P_{Inf}^{Pre}(s)$, and $P_{Inf}^{Sym}(s)$ were modelled from 77 transmission pairs obtained from publicly available sources within and outside mainland China (He et.al. 2020). Specifically, they estimated the serial interval to have a mean of 5.8 days based on a fitted gamma distribution. We used a gamma distribution with this mean, represented in Figure 1.

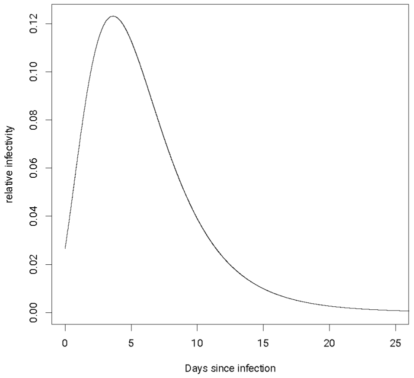

Figure 1. Gamma distribution used for the estimation.

For systematic cases we assumed an incubation period distribution of mean 5.2 days from a separate study of early COVID-19 cases (He et.al. 2020) and that infectiousness started from 2.3 days before symptom onset and peaked at 0.7 days before symptom onset and used a gamma distribution with these characteristics (See their Fig. 1c).

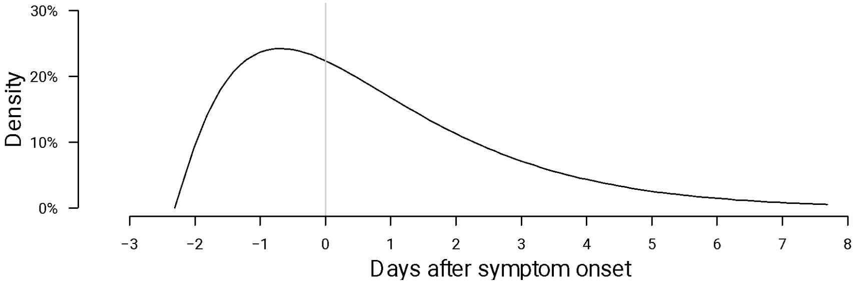

Figure 2. Gamma distribution for symptomatic cases.

Sensitivity Analysis for Modelling Assumptions.

It is possible that there is substantial population heterogeneity in people's infectiousness. Different infectiousness can be the result of age differences, differential immune system response, etc. We represent this as an individual specific multiplicative factor, λ_i, so that Y_i is modeled as Poisson with mean $\lambda_i R_0^A \int_{S_i}^{I_i} p_{inf}^{A_i}\left(t - S_i\right)dt$. We further model λ_i as random and independent between cases with mean 1 and standard deviation σ_{Het}. The case $\sigma_{Het} = 0$ corresponds to the special case of zero population heterogeneity in

infectiousness. We can interpret σ_{Het} as the typical percentage deviation of a person's infectivity from the average. Larger values of σ_{Het} reflect higher heterogeneity of infectiousness. We model the distribution of λ_i as Gamma$(1/\sigma^2_{Het}, 1/\sigma^2_{Het})$. This additional modeling adds a single new parameter, σ_{Het}, to the model. To fit the model, we continue to use the Bayesian framework with a prior for σ_{Het} being Gamma with mean 0.25 and standard deviation 0.25. The other aspects of the model are the same as reported in the paper.

Based on this model, the posterior mode of the basic reproduction number of asymptomatic cases is similar to that in the paper, that is, with $\sigma_{Het} = 0$. This more general model places somewhat more probability on larger values of R_0. The posterior mode for the standard deviation of the population heterogeneity, σ_{Het}, is about 1.0 indicating significant heterogeneity in reproductive number between cases.

We also changed the shape of the infectivity curve by changing the parameters of the gamma distributions to the upper and lower confidence bounds reported in He *et al* (2020). These did not substantively change the results.

Predicting the Epidemic Outcomes had Mitigation not been Applied

To better understand the potential impact this outbreak could have had in the absence of social distancing, quarantining, and other Covid-19 transmission mitigating measures, we implement an Susceptible-Infected-Recovered (SIR) model that outputs estimates of the prevalence, total number of cases, the incidence, number of new daily cases, and cumulative deaths. This SIR model is based on R_0^{Tot}. In an SIR model, there is a fixed sized population, and all members of the population are either Susceptible (not infected but can be infected), Infected (have the infection and can spread it to Susceptible people), or Recovered (had the infection but no longer have it and cannot spread it, nor can they get the infection again). We opt for an SIR model since the data collected only allows us to understand transmissions that occurred, but not contacts that didn't result in new cases. As a result, we lack information on exposure that can be credibly used in an SEIR model for this outbreak.

At any time t, suppose there are S susceptible, I infectious, and R recovered individuals. Denote the mean infectious time as $1/\gamma$ and recovery rate γ, for $\gamma > 0$, and the constant disease transmission rate β, so that βI is the rate of infection proportional to the number of infected, and βSI is the number of newly infected at each time t, proportional to the number of susceptible individuals. The SIR model is specified by the ODEs:

$$dS/dt = -\beta SI, \qquad dI/dt = \beta SI - \gamma I, \qquad dR/dt = \gamma I,$$

along with initial conditions $S(0) = S_0$, $I(0) > 0$, $I(0) << S(0)$, and $R(0) = 0$.

This model results in a reproductive number of $R_0 = \beta S_0/\gamma$. We assume a constant R_0 over the course of the outbreak, and we already have estimated a posterior R_0^{Tot} distribution. By taking many random draws from this distribution (100,000), we use the SIR model to estimate the prevalence, incidence, and cumulative deaths over time, along with best and worst case scenarios, over the course of the outbreak.
